# Sleep hygiene intervention for youth aged 10 to 18 years with problematic sleep: a before-after pilot study

**DOI:** 10.1186/1471-2431-12-189

**Published:** 2012-12-07

**Authors:** Evan Tan, Dione Healey, Andrew R Gray, Barbara C Galland

**Affiliations:** 1 Department of Women’s and Children’s Health, Dunedin School of Medicine, University of Otago, P.O Box 913, Dunedin, 9016, New Zealand; 2 Department of Psychology, University of Otago, Dunedin, New Zealand; 3 Department of Preventive and Social Medicine, University of Otago, Dunedin, New Zealand

**Keywords:** Sleep, Sleep hygiene, Sleep quality, Obesity, Accelerometry

## Abstract

**Background:**

The current study aimed to examine the changes following a sleep hygiene intervention on sleep hygiene practices, sleep quality, and daytime symptoms in youth.

**Methods:**

Participants aged 10–18 years with self-identified sleep problems completed our age-appropriate F.E.R.R.E.T (an acronym for the categories of Food, Emotions, Routine, Restrict, Environment and Timing) sleep hygiene programme; each category has three simple rules to encourage good sleep. Participants (and parents as appropriate) completed the Adolescent Sleep Hygiene Scale (ASHS), Pittsburgh Sleep Quality Index (PSQI), Sleep Disturbance Scale for Children (SDSC), Pediatric Daytime Sleepiness Scale (PDSS), and wore Actical® monitors twice before (1 and 2 weeks) and three times after (6, 12 and 20 weeks) the intervention. Anthropometric data were collected two weeks before and 20 weeks post-intervention.

**Results:**

Thirty-three youths (mean age 12.9 years; M/F = 0.8) enrolled, and retention was 100%. ASHS scores significantly improved (p = 0.005) from a baseline mean (SD) of 4.70 (0.41) to 4.95 (0.31) post-intervention, as did PSQI scores [7.47 (2.43) to 4.47 (2.37); p < 0.001] and SDSC scores [53.4 (9.0) to 39.2 (9.2); p < 0.001]. PDSS scores improved from a baseline of 16.5 (6.0) to 11.3 (6.0) post- intervention (p < 0.001). BMI z-scores with a baseline of 0.79 (1.18) decreased significantly (p = 0.001) post-intervention to 0.66 (1.19). Despite these improvements, sleep duration as estimated by Actical accelerometry did not change. There was however a significant decrease in daytime sedentary/light energy expenditure.

**Conclusions:**

Our findings suggest the F.E.R.R.E.T sleep hygiene education programme might be effective in improving sleep in children and adolescents. However because this was a before and after study and a pilot study with several limitations, the findings need to be addressed with caution, and would need to be replicated within a randomised controlled trial to prove efficacy.

**Trial registration:**

Australian New Zealand Clinical Trials Registry: ACTRN12612000649819

## Background

It is well established that children and adolescents require an average of at least nine hours of sleep per night [1-4] but unfortunately, it is also well known that children and adolescents are actually getting less than their recommended hours of sleep [2,3,5,6]. For example, Gibson and colleagues [6] found that 70% of students (aged 14–18 years) had less than eight and a half hours of sleep per night; with the average sleep time of the over 2000 students surveyed at about seven and a half hours. This inadequate sleep might be the reason why daytime sleepiness is prevalent in children and adolescents, with up to half of students surveyed in one study reporting daytime sleepiness at least once a week [7].

This pattern of inadequate sleep in children and adolescents is thought to be due to a combination of both intrinsic and extrinsic factors. Intrinsic factors include natural, developmental changes such as a shift in circadian rhythm during puberty [8], delayed sleep phase syndrome (estimated to occur in 7% of adolescents) [9], and sleep disordered breathing (SDB) such as obstructive sleep apnoea (OSA) [9,10]. However, insomnia type symptoms, such as difficulty falling asleep or staying asleep, seem to be the most prevalent cause of inadequate sleep with studies estimating insomnia type symptoms affecting up to 34% of adolescents [11,12]. Extrinsic factors, on the other hand, such as early school start times [13] and sleep habits (also known as sleep hygiene) such as caffeine consumption [14,15] and the use of electronic devices [16-18] near bedtime have also been shown to adversely impinge on adolescent sleep time. Indeed, numerous studies have shown good sleep hygiene to be an important predictor of sleep quality in adolescents [19-22]. As such, sleep hygiene has been used to try and alleviate the sleep problems found in adolescents today. For example, a behavioural sleep programme, incorporating sleep hygiene and stimulus control instructions was developed and trialled in University students in a bid to improve sleep quality [20]. A similar study also looked at the effectiveness of a school-based intervention in increasing sleep knowledge and improving adolescent sleep problems in high school students [23]. More interestingly, a behavioural sleep intervention utilizing a combination of sleep hygiene, cognitive therapy and stress reduction techniques has also been used as a means for improving sleep in the hope of lowering the risk for recidivism of substance abuse in adolescent substance users [24]. These sleep hygiene studies have reported positive outcomes in various aspects of sleep quality [20,23,24] and even drug use at 12 months follow-up [24]. However, these studies have focussed on older aged adolescents and were delivered in a group or classroom setting. We have taken a different approach and developed a one-on-one sleep hygiene programme designed specifically for children and adolescents aged 10–18 years old. To the best of our knowledge, this sleep programme is the first of its kind to be developed for youth in consultation with children and adolescents themselves. The current study thus aimed to examine the changes following a novel sleep hygiene intervention on sleep hygiene, sleep quality, and daytime symptoms in children and adolescents. As a secondary outcome, the study also measured changes in BMI because of reported associations between inadequate sleep, disturbed sleep and overweight and obesity in children and adolescents [10,25].

## Methods

### Development of the intervention

This was developed prior to the pilot study [26]. Based on a review of the literature, the researchers assembled two thematic versions (animal theme, sports theme) of a sleep hygiene education programme covering three main sleep hygiene categories of sleep routine, sleep environment and eating and drinking habits before bedtime. Twenty-two developmentally healthy children and adolescents of the same age group as the pilot population were recruited and consulted about the programmes; 11 aged 10 to 14 years (5 females, 6 males) and 11 aged 15 to 18 years (6 females, 5 males). Both thematic versions of the programmes were delivered (alternate sequences) by the researcher to participants (in pairs), and at the conclusion, participants completed a pop quiz to evaluate recall and were then interviewed about the suitability of content structure (including wording), programme preference, and their ideas to aid compliance to the programme. Interviews were transcribed and naive reading and thematic structural analyses [27] conducted on the transcripts. Results showed the animal theme version had the highest correct acronym and rule recall (96% and 27% animal theme respectively versus 18% and 23% sports theme respectively) and was equally appealing to both boys and girls and across both age groups. Based on the interview results, wording was modified and aesthetic changes made to the multi-media presentation and take-home resource materials. The final programme was packaged as the F.E.R.R.E.T sleep hygiene programme; an acronym for Food, Emotions, Routine, Restrict, Environment and Timing, with each category consisting of three easy to remember sleep hygiene rules pertaining to their respective categories with the last rule reinforcing the acronym (Table 1). Explanations of rules and how best to implement them were covered within the education and resource materials.

**Table 1:** Three main rules pertaining to the six themes of the F.E.R.R.E.T study

**Theme**	**Rule 1**	**Rule 2**	**Rule 3**
Food	Don’t drink anything 30 minutes before bed	Stay away from food and caffeine 3 hours before bed	No alcohol or smoking 3 hours before bed
Emotions	Set a time during the day for things you want to think about or plan for	Wind down and relax 30 minutes before bed	Try not to worry, think about things or plan things in bed
Routine	Wake up and go to bed the same time every day	Bring light into your room when you wake up and dim lights before bed	Your sleep routine should be kept the same each day
Restrict	No electronic media (e.g. iPods, TV watching) at least 30 minutes before bed	No exercise 3 hours before bed	Don’t do anything else in bed except sleep (e.g. no homework)
Environment	You should be comfortable in your pyjamas and bedroom	Control light, temperature and noise	Keep clocks faced away from your bed
Timing	Try not to sleep more or less than your recommended hours of sleep	The rules have been kept at 30 minutes before bed or 3 hours for you to remember them easily	Try to stick to the rules

### Pilot of the intervention: participant recruitment

Participants were recruited, from the community via newspaper advertisements, posters (placed in supermarkets and the local hospital), letters to schools, and by word of mouth. Inclusion criteria were children/adolescents aged 10–18 years who experience difficulties falling asleep, and/or maintaining sleep. Exclusion criteria included current diagnosis of a primary sleep disorder or any uncontrolled medical condition, living outside the greater Dunedin area, and taking prescribed or over-the-counter medication for insomnia currently, or throughout the period of enrolment. An initial phone call was made after the participants had volunteered to ensure eligibility and then assessed again at the first face-face appointment. This study was approved by the Lower South Regional Ethics Committee, Dunedin, New Zealand (Project Key: LRS/07/12/055). Information sheets were sent out and written informed consent obtained, from both parent and child prior to commencement of the study.

### Intervention delivery

All participants recruited in the study received the sleep hygiene education programme delivered, in a private one-one session to the participant and one of their parents by the principal investigator. During the intervention session lasting approximately 90 minutes, participants were also given an individualised F.E.R.R.E.T flip-book containing the acronyms and rules in an easy reference format, and space to write notes down should they wish to. The F.E.R.R.E.T flip-book also had a compact disc (CD) containing the F.E.R.R.E.T multi-media presentation and participants were also given a number of resources to help them remember what they had learnt during the intervention: a wall poster, fridge magnets and a progress chart. Post-intervention support included fortnightly telephone calls to the participant, a dedicated F.E.R.R.E.T cell phone number that participants could call or text at any time with sleep issues, as well as one-one informal session every six weeks with the researcher to discuss progress.

### Data collection

Table 2 gives the data collection tools for main and secondary outcomes (described below) and time-points for each. Sleep questionnaires were completed at two time points pre-intervention (1 and 2 weeks prior) and 6, 12 and 20 weeks post-intervention. Anthropometric measures were taken once pre-intervention (2 weeks) and once post intervention (20 weeks post-intervention). In preparation for the first data collection point (2 weeks pre-intervention), sleep questionnaires and Acticals were delivered the week prior. Participants then brought the completed questionnaires and Actical® monitors to this first visit. Anthropometric measures were taken and a parent/guardian completed the demographic questionnaire covering participant age, ethnicity, and residential address, and parental level of education and household income. Deprivation index values were generated based on the NZDep2006 value from the participants’ given addresses where 1 represents the areas with the least deprived scores and 10, the areas with the most deprived scores. The deprivation index is measured from nine characteristics related to deprivation such as telephone and automobile access, household income and occupant’s educational qualifications.

**Table 2:** Data collection tools and time points for each

**Measures**	**Collection Tools**	**Intervention (weeks)**				
		**Pre-**		**Post-**		
		**2**	**1**	**6**	**12**	**20**
Socio-demographic	Self-styled questionnaire	✓	-	-	-	-
Anthropometric	Height, weight, waist circumference	✓	-	-	-	✓
Sleep hygiene	Adolescent Sleep Hygiene Scale	✓	✓	✓	✓	✓
Sleep quality	Pittsburgh Sleep Quality Index	✓	✓	✓	✓	✓
Sleep quality (parent report)	Sleep Disturbance Scale for Children	✓	✓	✓	✓	✓
Daytime sleepiness	Pediatric Daytime Sleepiness Scale	✓	✓	✓	✓	✓
Sleep duration & energy expenditure	Actical	✓	✓	✓	✓	✓

### Main outcome measures

#### Adolescent sleep hygiene scale (ASHS)

The ASHS is a 28-item self-report questionnaire that assesses sleep-facilitating and sleep-inhibiting practices in adolescents along nine different domains: physiological, cognitive, emotional, sleep environment, daytime sleep, substances, bedtime routine, sleep stability, and bed/bedroom sharing [28]. Participants report sleep habits practiced along a 6-point scale with an overall sleep hygiene score (internal consistency, Cronbach’s α = 0.80) obtained from the mean of the domain scores with higher scores indicative of better sleep hygiene [28]. The ASHS shows concurrent validity associations with the Adolescent Sleep Wake Scale (ASWS) [28], and was rated as “approaching well-established” in a recent review [29]. Furthermore, the ASHS is also the only sleep hygiene measure appropriate for use with children older than 12 years of age [29].

#### Pittsburgh sleep quality index (PSQI)

The PSQI is a 19-item self-report questionnaire that measures sleep quality during the previous month to discriminate between good and poor sleepers [30]. The PSQI generates seven domain scores with each domain score ranging from zero to three. The domain scores are summed to produce a global score ranging from zero to 21, where a PSQI global score of greater than five is considered to be suggestive of significant sleep disturbance. In adults, the PSQI has good reliability with high internal consistency (α = 0.83) as well as test-retest reliability (*r* = 0.85) [30].

#### Sleep Disturbance Scale for Children (SDSC)

The SDSC is a 26-item parent-report questionnaire which measures sleep quality and disturbance in children and adolescents. The SDSC has high internal consistency (α = 0.79), test/retest reliability (*r* = 0.71) [31] and has been shown to be effective at assessing sleep disturbances in a similar age group as the present study [32,33].

#### Pediatric daytime sleepiness scale (PDSS)

The PDSS is an eight item, self report questionnaire which measures daytime sleepiness in school age populations, with possible scores ranging from 0–32 with higher PDSS scores indicating greater daytime sleepiness. Aside from being easy to administer, score and interpret, the PDSS has high internal consistency (α) of at least 0.80 in split-half samples and acceptable factor loadings (> 0.4) [34].

### Secondary outcome measures

#### Anthropometric measures

Height and weight measurements were carried out at the children’s outpatient clinic of the Dunedin Public Hospital and BMI and BMI z-scores calculated. Height, to the nearest 0.1 cm, was measured using a wall-mounted stadiometer and weight, to the nearest 0.1 kg, was measured with shoes, jackets, sweatshirts and hats removed. Waist circumference (WC) was also measured, to the nearest 0.1 cm, at the level of the iliac crest just below the navel.

### Accelerometry: physical activity and sleep

These data were collected at baseline and 20 weeks post-intervention. Physical activity over a seven day period was measured using an Actical® accelerometer (Mini-Mitter, Respironics, USA). The Actical accelerometer is a small, water resistant motion sensor which is capable of sensing and recording omnidirectional acceleration and activity ranging from sedentary to vigorous movements [35]. Participants wore the Actical accelerometer, which was attached to an elastic belt, on their hips. The Actical accelerometer is most sensitive to vertical movements of the trunk [36] and hip placement has been shown to provide the most accurate movement measurements [37]. Energy expenditure was calculated by converting activity counts into calories based on the participants’ weight. Estimates of sleep duration were also obtained using a zero-count threshold. Although the Actical is traditionally used as a core based accelerometer to measure physical activity, Weiss et al. [38] have evaluated the use of Acticals to estimate sleep in adolescents. The authors concluded that the Actical provided measures of sleep duration comparable in accuracy to other sleep-wake estimation wrist devices, as well as from PSG.

### Power analysis

As this was a pilot study, we did not power on changes of key outcomes but rather on the reliability of measures used with the intention that this would assist with the design of a large-scale RCT. Sample size calculations indicated that 22 participants were required to provide 80% power when using a two-sided test at the 0.05 level to show that reliability of continuous measures as assessed by the ICC was greater than 0.60 (indicating good reliability) assuming a true reliability of at least 0.85 (very good). Further, a sample size of 28 participants would be sufficient in the same way for showing that reliability of categorical measures, as assessed by Kappa, was at least 0.60 (indicating at least adequate reliability) assuming a true Kappa of 0.85 (very good) or better and 50% responses in each category. As missing data was expected to be minimal, a final sample size of 33 was chosen.

### Statistical analysis

For the questionnaire and Actical® data, the mean of week *−*2 and week *−*1 were used as estimates of baseline values i.e. pre-intervention. Using baseline values and data from weeks 6, 12, and 20, random coefficient models with random participant effects and random slopes were constructed for each outcome of interest. Sex, BMI (pre-intervention), and age (pre-intervention) were controlled for in all models. Fractional polynomial regression (using Huber-White estimators to adjust the standard errors for the repeated measures on each participant) was used to identify and where evident to model non-linear associations over time. For anthropometric measures, with just one pre- and one post-intervention measure available, paired t-tests were used. Statistical analyses were performed using Stata version 11.0 (StataCorp, College Station, TX). All tests were two-sided with p < 0.05 indicating statistical significance.

## Results

### Participant demographic characteristics and baseline sleep

Thirty-three participants, with self-reported sleep problems, enrolled in the study and their demographic characteristics are summarized in Table 3. The retention rate was 100% with all participants completing the study. Baseline mean (SD) sleep quality data obtained via the PSQI and SDSC were 7.47 (2.43) and 53.4 (9.0) respectively and were values indicative of poor sleep quality [30,31]. Baseline mean (SD) daytime sleepiness scores of 16.5 (6.0) were also elevated and indicative of excessive daytime sleepiness [34,39]. However, baseline mean (SD) sleep duration of 552 (58) minutes obtained by Actical® plots was within the recommended sleep times for this age group [1-3].

**Table 3:** Demographic characteristics of the study participants

**Characteristics (n = 33)**	
Mean age in years (SD)	12.9 (2.19)
Sex	
Male	15 (45%)
Female	18 (55%)
Ethnicity	
European	28 (85%)
Māori	3 (9%)
Asian	1 (3%)
Other	1 (3%)
Deprivation Index; mean (SD)	4.27 (2.45)
Maternal Education	
Tertiary	18 (56%)
High School	13 (41%)
None	1 (3%)

### Changes in outcome measures between baseline and 20 weeks follow-up *Anthropometry*

There was a small but statistically significant reduction in BMI z-scores (Table 4). Mean (SD) BMI z-scores significantly decreased (p = 0.001) approximately 16 percent 20 weeks post-intervention from 0.79 (1.18) to 0.66 (1.19). No significant differences were seen in waist circumference before and after intervention.

**Table 4:** Change in anthropometry, Actical and sleep measures between baseline and 20 weeks

	**n**	**Baseline**	**20 weeks**	**Δ Mean**	**P value**
		**Mean (SD)**	**Mean (SD)**	**(95% CI)**	
**Anthropometric Measures**					
Weight (kg)	33	55.8 (16.1)	57.4 (15.8)	1.61 (−1.02 to 2.21)	<0.001
Height (m)	33	1.59 (0.13)	1.62 (0.12)	0.03 (0.02 to 0.03)	<0.001
Height z-score	33	0.50 (1.06)	0.56 (1.02)	0.06 (−0.03 to 0.15)	0.158
BMI (kg/m^2^)	33	21.7 (4.50)	21.7 (4.49)	−0.06 (−0.28 to 0.16)	0.607
BMI z-score	33	0.79 (1.18)	0.66 (1.19)	−0.13 (−0.20 to 0.05)	0.001
Waist Circumference (cm)	32	78.9 (11.8)	78.3 (15.5)	−0.64 (−3.55 to 2.28)	0.659
**Sleep Questionnaires**					
ASHS (Sleep Hygiene)	32	4.70 (0.41)	4.95 (0.31)	0.19 (0.06 to 0.33)	0.005
PSQI (Self report sleep quality)	33	7.47 (2.43)	4.47 (2.37)	−2.93 (−3.61 to −2.24)	<0.001
SDSC (Parent report sleep quality)	32	53.4 (9.0)	39.2 (9.2)	−14.1 (−17.1 to −11.1)	<0.001
PDSS (Daytime sleepiness)	33	16.5 (6.0)	11.3 (6.0)	−5.1 (−7.0 to −3.2)	<0.001
**Actical® measures**					
Sleep Duration (minutes)	32	552 (58)	557 (63)	−1 (−19 to 16)	0.906
Sleep total ee^a^	32	18.5 (54.8)	5.5 (6.3)	−11.9 (−25.7 to 1.9)	0.090
Day total ee	32	568 (224)	533 (263)	−38 (−119 to 43)	0.356
Day sedentary/light ee	32	238 (90)	214 (80)	−27 (−50 to −3)	0.025
Day moderate/vigorous ee	32	330 (152)	319 (206)	−12 (−80 to 57)	0.740

^a^Energy expenditure in kilocalories.

Means shown are unadjusted. Baseline data represent the mean of the two pre-intervention measures for sleep questionnaires and Actical data, and the one pre-intervention measure for anthropometry. Changes in means and p-values were determined from paired t-tests for anthropometric and Actical measures and using regression for sleep measures. For sleep measures, changes are determined as the change in the slope of the regression curves over 20 weeks.

#### Sleep measures

**Figure 1:**
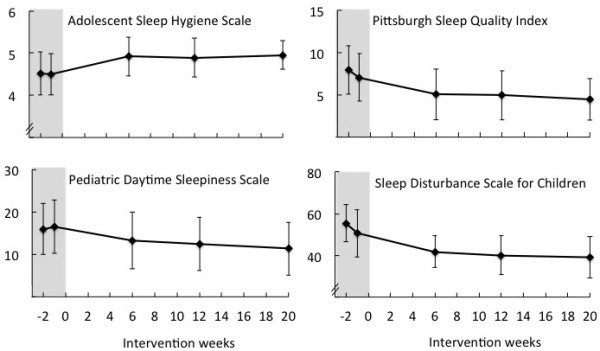
**Sleep questionnaire data (mean ± SD) plotted by time point in relation to intervention.** Intervention occurred at time 0 weeks. Shaded areas represent pre-intervention time points.

Significant improvements were observed across all sleep questionnaire measures (Table 4 and Figure 1). Mean (SD) sleep hygiene (ASHS) scores, increased significantly (p = 0.005) from 4.70 (0.41) to 4.95 (0.31) 20 weeks post-intervention. Mean (SD) self-reported sleep disturbance scores (PSQI) reduced significantly (p < 0.001) from 7.47 (2.43) to 4.47 (2.37) post-intervention, as did mean (SD) parent-reported sleep disturbance scores (SDSC) which decreased from a mean of 53.4 (9.0) to 39.2 (9.2) (p < 0.001). Self-reported mean (SD) scores of daytime sleepiness also decreased significantly post-intervention from 16.5 (6.0) to 11.3 (6.0) (p < 0.001).

#### Actical measures

No significant differences were seen in Actical measures after the sleep hygiene intervention except for a decrease in sedentary/light energy expenditure (p = 0.025) during daytime (Table 4). Mean (SD) energy expenditure from sedentary/light activity decreased from 238 (90) to 214 (80) kilocalories. However, no significant changes were seen in moderate/vigorous activity (p = 0.740). A tendency for reduced energy expenditure during sleep (p = 0.090) was seen 20 weeks post-intervention although individual energy expenditure during sleep (sedentary/light or moderate/vigorous) could not be analyzed due to clear heteroscedasticity which could not be stabilised due to zero values.

## Discussion

The current study aimed to examine the changes following a newly developed sleep-hygiene intervention in relation to sleep hygiene practices, sleep quality, and daytime symptoms in children and adolescents. We found significant improvements in sleep hygiene, sleep quality as well as daytime symptoms such as a reduction in daytime sleepiness and sedentary/light activity, following the intervention. We reported statistically significant improvements in sleep hygiene practices post-intervention which earlier studies have also demonstrated [20,24,40]. The improvements in sleep practices (as assessed by the ASHS) reported in the current study, albeit significant, were small. However, although the improvements in sleep practices reported are slight, the statistically significant increase might indeed reflect real-life benefits as seen in the improvements in sleep quality reported by both parents and participants, as well as the decrease in daytime sleepiness.

The improvements in sleep hygiene and sleep quality reported in the current study are also consistent with previous studies that have shown similar findings in participants ranging from infants to adults [21,24,40-42]. However given both participants and parents were aware of the treatment, a study of sleep hygiene intervention with a concurrent control group with parents blind to treatment would be required to provide definitive answers in regard to efficacy. Baseline data on sleep quality and daytime sleepiness indicated that the cohort of enrolled children had poor sleep quality and elevated index scores for daytime sleepiness. With regard to sleep quality, baseline mean (SD) PSQI scores of 7.47 (2.43) were greater than five, indicating poor sleep quality [30] while baseline mean (SD) SDSC scores of 53.4 (9.0) were greater than 39 which was also an indication of poor sleep quality [31]. These scores were very similar to those reported in slightly older students with poor sleep quality [20,43] and baseline SDSC index scores were also very similar to those of patients with known sleep disorders such as insomnia and respiratory disturbances [31]. Baseline daytime sleepiness scores were also indicative of daytime sleepiness [34,38] and similar to those reported in children of a similar age with OSA and epilepsy [44]. The baseline mean sleep duration was within recommended sleep times for this age group [1-3] which might be why the mean increase in total sleep time (after the intervention) was only five minutes.

A significant decrease in daytime sleepiness (PDSS), a daytime symptom of poor sleep quality, also suggests further evidence of improved sleep quality. Mean scores for PSQI, SDSC, and PDSS improved from scores indicative of sleep dysfunction or above control group means, to acceptable or control group mean scores. There was a trend (albeit non-significant) for energy expenditure during sleep to decrease post-intervention which could indicate this as a marker for the improved sleep quality reported subjectively. Furthermore, a significant decrease in participants’ sedentary/light activity behaviour during the day was also observed which could be due to the decrease in sleepiness during the day as observed in PDSS scores.

Encouragingly, we found a slight but significant reduction in BMI z-scores 20 weeks post-intervention. This seems to suggest that although significant increases in absolute weight and height were also observed, the reduction in BMI z-scores reflects a slower weight gain of our participants (compared to peers of the same age and sex) rather than an increase in height that led to improved BMI z-scores. The recruited sample of children, although not overweight, had BMI z-scores almost one SD above the population mean that reduced by *−*0.13 (CI *−*0.20 to 0.05, p = 0.001) to a mean (SD) BMI z-score of 0.66 (1.19) 20 weeks post-intervention. An earlier study, adopting a diet and physical activity behavioural intervention in children [45] reported a slightly smaller reduction in BMI z-scores, as did a similar study in obese adolescents [46]. Compared to the two above mentioned studies, the reduction in BMI z-scores reported in the current study are important given the fact that our sample of children were not overweight/obese. It is possible improvements in sleep might have led to the decrease in BMI z-scores seen through the changes in the regulation of appetite hormones leptin and ghrelin [47-49] and/or due to changes in fat metabolism [50]. However caution must be applied in linking this BMI reduction to improved sleep as rules around food restriction before bedtime were part of the sleep hygiene practice regime to encourage sleep and thus more research is needed to corroborate our findings around sleep and weight. A larger, behavioural sleep study investigating the health outcomes of obese adults after a behavioural sleep extension intervention of sleep deprived (< 6.5 hours) participants is currently underway [51]. It employs a non-pharmacological sleep only intervention (no modifications to energy intake or expenditure). Interim analysis of PSQI scores also revealed a greater reduction in PSQI scores in the sleep intervention group compared to the control group at the first year of follow-up [51]. These trends provide indications that sleep may be an effective adjunct treatment strategy for weight management.

This study was not without limitations. First, our sample was a small community-recruited sample, which limits generalizability. For example, as enrolment to the study was voluntary and dependent on the participant/family approaching us, this cohort of participants could have been especially motivated. Secondly, this was a before and after intervention study and did not include a control group, thus any improvements seen could be due to the placebo effect which might have been amplified by the participants’ motivation for change. Thirdly, although the measurement of sleep quality by participant using the PSQI is written in a language easily understood by this age group, the validity and reliability of the instrument in this young age group has yet to be demonstrated. Finally, it is likely that the self-selected recruitment of parents will have produced a sample of children with high levels of sleep problems. This was an intended effect of the recruitment strategies used in order to demonstrate that the pilot was acceptable for this population and so could be used in a larger trial. In the absence of a control group, however, regression towards the mean cannot be ruled out as at least partially explaining the changes observed during the study. The full study was completed over a school year, thus we cannot exclude seasonal changes being an advantage or disadvantage to sleep changes in some. A strength of the study was the retention rate at 100% with every enrolled participant completing the 20 week F.E.R.R.E.T sleep hygiene programme. This is probably atypical, but the high retention may have reflected the age-appropriateness of the intervention, perhaps aided by the multiple researcher contact time points and/or by the individuals being more motivated to complete because they volunteered and presented with self-identified sleep problems.

## Conclusions

Our findings suggest the F.E.R.R.E.T sleep hygiene education programme might be effective in improving sleep in children and adolescents, but given the limitations of a pilot study, caution must be applied in interpretation of the findings. The weight loss findings support the current research impetus on the link between sleep and obesity. However because this was a before and after study within a pilot study, the findings would need to be replicated within a randomized controlled trial to prove efficacy, and ideally within a sleep hygiene intervention that does not address pre-bedtime food consumption. Future research should also investigate the long-term effect of a sleep hygiene intervention on various physiological, psychological, and behavioural health outcomes in youth.

## Abbreviations

ASHS: Adolescent sleep hygiene scale; PDSS: Pediatric daytime sleepiness scale; PSQI: Pittsburgh sleep quality index; SDSC: Sleep disturbance scale for children.

## Competing interests

The authors declare that they have no competing interests.

## Authors’ contributions

The study chief investigators ET and BCG were responsible for identifying the research question, study design, obtaining ethical approval, overseeing the day-to-day implementation of the study, and securing funding. DMH provided expertise in intervention development and data interpretation. ARG conducted the statistical analysis of all data. All authors read and approved the final manuscript.

## Pre-publication history

The pre-publication history for this paper can be accessed here:

http://www.biomedcentral.com/1471-2431/12/189/prepub
